# Specific detection of OCT3/4 isoform A/B/B1 expression in solid (germ cell) tumours and cell lines: confirmation of OCT3/4 specificity for germ cell tumours

**DOI:** 10.1038/bjc.2011.270

**Published:** 2011-08-16

**Authors:** M A Rijlaarsdam, H A D M van Herk, A J M Gillis, H Stoop, G Jenster, J Martens, G J L H van Leenders, W Dinjens, A M Hoogland, M Timmermans, L H J Looijenga

**Affiliations:** 1 Department of Pathology, Josephine Nefkens Institute, Daniel den Hoed Cancer Center, Rotterdam, The Netherlands; 2 Department of Urology, Erasmus MC-University Medical Center Rotterdam, Josephine Nefkens Institute, Daniel den Hoed Cancer Center, Rotterdam, The Netherlands; 3 Department of Internal Medicine, Josephine Nefkens Institute, Daniel den Hoed Cancer Center, Rotterdam, The Netherlands

**Keywords:** OCT3/4, isoforms, germ cell tumour, somatic cancers, mRNA expression

## Abstract

**Background:**

OCT3/4 (POU5F1) is an established diagnostic immunohistochemical marker for specific histological variants of human malignant germ cell tumours (GCTs), including the seminomatous types and the stem cell component of non-seminomas, known as embryonal carcinoma. OCT3/4 is crucial for the regulation of pluripotency and the self-renewal of normal embryonic stem- and germ cells. Detection of expression of this transcription factor is complicated by the existence of multiple pseudogenes and isoforms. Various claims have been made about *OCT3/4* expression in non-GCTs, possibly related to using nonspecific detection methods. False-positive findings undermine the applicability of OCT3/4 as a specific diagnostic tool in a clinical setting. In addition, false-positive findings could result in misinterpretation of pluripotency regulation in solid somatic cancers and their stem cells. Of the three identified isoforms – *OCT4A*, *OCT4B* and *OCT4B1* – only OCT4A proved to regulate pluripotency. Up until now, no convincing nuclear OCT4A protein expression has been shown in somatic cancers or tissues.

**Methods:**

This study investigates expression of the various OCT3/4 isoforms in GCTs (both differentiated and undifferentiated) and somatic (non-germ cell) cancers, including representative cell lines and xenografts.

**Results:**

Using specific methods, *OCT4A* and *OCT4B1* are shown to be preferentially expressed in undifferentiated GCTs. The *OCT4B* variant shows no difference in expression between GCTs (either differentiated or undifferentiated) and somatic cancers. In spite of the presence of OCT4A mRNA in somatic cancer-derived cell lines, no OCT3/4 protein is detected. Significant positive correlations between all isoforms of OCT3/4 were identified in both tumours with and without a known stem cell component, possibly indicating synergistic roles of these isoforms.

**Conclusion:**

This study confirms that OCT4A protein only appears in seminomatous GCTs, embryonal carcinoma and representative cell lines. Furthermore, it emphasises that in order to correctly assess the presence of functional OCT3/4, both isoform specific mRNA and protein detection are required.

OCT3/4 (also known as POU5F1) is a well-known marker for pluripotent stem cells, both physiologically and artificially induced (Pesce and Scholer, 2000; Wang and Dai, 2010). In addition, it is also expressed in primordial germ cells (PGCs) (Pesce and Scholer, 2000), the stem cell of gametogenesis later in life. OCT3/4 is a transcription factor involved in self-renewal and pluripotency (Niwa *et al*, 2000; Pesce and Scholer, 2001), and might counteract apoptosis in PGCs (Kehler *et al*, 2004). During further development (differentiation/maturation) of these types of embryonic cells, expression is downregulated and finally lost in the differentiated derivatives. Owing to this specific pattern of expression during embryogenesis, which is retained during the process of malignant transformation, OCT3/4 is an established and highly informative diagnostic marker for defined types of malignant germ cell tumours (GCTs), especially those of the seminomatous cell type (seminoma (SE), dysgerminoma and germinoma) and embryonal carcinoma (Looijenga *et al*, 2003; de Jong and Looijenga, 2006; Cheng *et al*, 2007; Looijenga, 2009).

The *OCT3/4* gene is located on human chromosome 6 band p21 and consists of five exons (Takeda *et al*, 1992; Krishnan *et al*, 1995). It encodes a protein belonging to the family of octamer-binding proteins that specifically binds to the conserved ATTTTGCAT motive in transcriptional control elements of genes. This sequence is recognised by the highly charged POU domain of the OCT3/4 protein, explaining its alternative name. The POU domain consists of two subdomains: a C-terminal homeodomain and an N-terminal POU-specific region separated by a short non-conserved linker (Sturm and Herr, 1988).

Various investigations of OCT3/4 expression are reported, both on mRNA and protein level. Most are complicated by the existence of pseudogenes and splice variants (isoforms) (de Jong and Looijenga, 2006; Liedtke *et al*, 2008; Wang and Dai, 2010), possibly leading to findings of false-positive expression. Till date, five OCT3/4 pseudogenes have been identified. These will be amplified by most of the published primer sets, owing to their high sequence similarity to OCT4A (Pain *et al*, 2005; Suo *et al*, 2005). However, proper DNase pre-treatment of the samples will exclude this technical artefact, which is a rather simple and straightforward step to include in the experimental setup.

In addition, three splice variants (isoforms) of *OCT3/4* have been identified (Wang and Dai, 2010). The best-known isoform is referred to as OCT4A. This variant is reported to be stem cell-specific, whereas the function(s) of the other two variants, that is, OCT4B and OCT4B1, are still under investigation (Gao *et al*, 2010; Wang and Dai, 2010; Asadi *et al*, 2011). However, it has been demonstrated that OCT4B is unable to activate or repress transcription of known OCT4A-responsive genes (Lee *et al*, 2006). Therefore, OCT4B seems unlikely to be directly involved in transcriptional regulation of pluripotency and self-renewal. OCT4B1 on the other hand, has recently been suggested to have a role in both regulation of pluripotency (Atlasi *et al*, 2008; Papamichos *et al*, 2009; Asadi *et al*, 2011) and OCT4B-mediated functions (Gao *et al*, 2010).

As indicated, OCT3/4 has mainly been linked to pluripotency, for which it is a well-known and established marker. Pluripotency refers to the capacity of a (embryonic) stem cell to generate all different tissues (endo-, ecto- and mesodermal differentiation). Indeed, differentiation induction is associated with downregulation of OCT3/4 (Botquin *et al*, 1998; Velkey and O’Shea, 2003; Hay *et al*, 2004; Matin *et al*, 2004; Zaehres and Scholer, 2007). The other way around, OCT3/4 downregulation results in loss of stem cells and induction of differentiation (Niwa *et al*, 2000). A high-throughput immunohistochemical screen of many different types of human cancers demonstrated that OCT3/4 is a specific and highly informative diagnostic marker for seminomatous tumours, which are the malignant counterparts of PGCs/gonocytes, as well as embryonal carcinomas, the stem cell component of non-seminomas (Looijenga *et al*, 2003; de Jong *et al*, 2005). This observation is confirmed by multiple independent studies, as reviewed before (Looijenga, 2009). The overall findings resulted in the conclusion that OCT3/4 is an excellent, and currently successfully used, diagnostic marker for the detection of undifferentiated variants of so-called type II GCTs (UNDIF-GCTs=SE or dysgerminoma/germinoma, and the stem cell component of non-seminoma (NS, specifically embryonal carcinoma)), as well as their precursor stages (carcinoma *in situ* of the testis (CIS) and gonadoblastoma of dysgenetic gonads) (Looijenga *et al*, 2003; de Jong *et al*, 2005, 2008b; de Jong and Looijenga, 2006; Cheng *et al*, 2007). Most recently, OCT3/4 protein detection has been used as a diagnostic tool for the non-invasive diagnosis of CIS (van Casteren *et al*, 2008).

In non-GCTs (N-GCTs), a highly heterogeneous expression pattern of OCT3/4, both mRNA and protein, is reported. This might be due to the use of nonspecific primers detecting other isoforms, improper DNAse pretreatment (resulting in amplification of pseudogenes) and incorrect interpretation of immunohistochemical stainings. The results of the various articles (Ezeh *et al*, 2005; Tai *et al*, 2005; Atlasi *et al*, 2007; Lengner *et al*, 2007; Chen *et al*, 2008; Lai *et al*, 2009; Sotomayor *et al*, 2009) to whether or not there is expression of (functional) *OCT3/4* in solid cancers are therefore inconclusive. To further investigate this issue, the current study was undertaken. It investigates expression of the different isoforms of *OCT3/4* mRNAs and protein in various types of solid cancers: undifferentiated GCTs (UNDIF-GCTs) and GCTs without an embryonic stem cell component (DIF-GCTs=yolk sac tumours and teratomas) as well as N-GCTs. Also, representative cell lines (−CL) of UNDIF-GCT and N-GCT are included.

## Materials and methods

### Materials

The cell lines and tumour samples included in this study are indicated in Table 1. All samples/cell lines were obtained from different Departments in the Josephine Nefkens Institute (Erasmus MC-University Medical Center, the Netherlands). The prostate carcinoma cell lines and xenografts are extensively reviewed elsewhere (van Weerden *et al*, 1996; Marques *et al*, 2006). This also goes for the GCT-CLs (Andrews *et al*, 1996; de Jong *et al*, 2007, 2008a).

### RNA isolation

**Figure 1:**
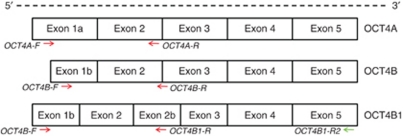
mRNA structure of the different *OCT3/4* splice variants. All *OCT3/4* splice variants have a similar 3′ ends but differ in their 5′ start (i.e., exons 1a and 1b). Moreover *OCT4B1* includes a previously identified intronic region now named exon 2b. For a detailed discussion see Atlasi *et al*, 2008. Red arrows indicate forward (−F) and reverse (−R) primers from (Atlasi *et al*, 2008) used in the reverse transcription–polymerase chain reaction to specifically identify the different *OCT3/4* splice variants. Green arrow denotes a specific reverse primer used to sequence *OCT4B1*. The colour reproduction of this figure is available at the *British Journal of Cancer* online.

High-quality total RNA was extracted from the above mentioned cell lines and tumour samples using TRIzol Reagent (Invitrogen, Breda, The Netherlands) according to the manufacturer's instructions. Samples were pretreated with DNase I, checked for residual DNA contamination by PCR, after which cDNA synthesis was performed as described before (Looijenga *et al*, 2006; de Jong *et al*, 2008a). For each sample, a no-reverse transcription control was used, and *HPRT* was used as reference level of expression. Quantitative PCR was performed using the Real-Time PCR HT7900 (Applied Biosystems, Foster City, CA, USA). Sequences for the OCT3/4 splice variant specific primers were as described before (Atlasi *et al*, 2008; de Jong *et al*, 2008a). These are highly specific for the different isoforms and even discriminate between OCT4A and its pseudogenes. The following forward (−F) and reverse (−R) primers were used (annotation between brackets=annotation from (Atlasi *et al*, 2008)): HPRT: HPRT244-exon2-F, 5′-AATTATGGACAGGACTGAACGTC-3 ′ HPRT243-exon3-R, 5′-CGTGGGGTCCTTTTCACCAGCAAG-3′. OCT4A: OCT4A-F (OCT4-AF) 5′-CTTCTCGCCCCCTCCAGGT-3 ′ OCT4A-R (OCT4-RB1) 5′-AAATAGAACCCCCAGGGTGAGC-3 ′. OCT4B: OCT4B-F (OCT4-FB) 5′-AGACTATTCCTTGGGGCCACAC-3′ OCT4B-R (OCT4-RB5) 5′-GGCTGAATACCTTCCCAAATAGA-3. OCT4B1: OCT4B-F (OCT4-FB), 5′-AGACTATTCCTTGGGGCCACAC-3′ OCT4B1-R (OCT-RB4) 5′-CCCCCTGTCCCCCATTCCTA-3′. The localisation of the different primers is depicted in Figure 1. The efficiency and specificity of these primers was extensively tested before (Atlasi *et al*, 2008). The specificity for human RNA is proven by the absence of any *OCT4*A/B/B1 expression in most of the xenografts, specifically in PC82, which has a large stromal component. Quantitative values were obtained from the *C*_t_. *OCT3/4* mRNAs (A, B and B1) were quantified with relative to *HPRT* (*OCT3/4* mRNA=2 ^(mean *C*_t_^ ^HPRT−mean *C*_t_^ ^OCT3/4 (A, B or B1)^) as described before (Livak and Schmittgen, 2001). The *OCT4*B1 PCR-products were sequenced using OCT4B1-F and a primer in exon 5 (OCT4B1-R2: (OCT4-RB3) 5′-CCCCCTGTCCCCCATTCCTA-3′) to verify the nature of this splice variant. MicroRNA expression was measured as described previously (Gillis *et al*, 2007).

### Immunohistochemistry

Immunohistochemistry was performed on paraffin-embedded tissue sections of 4 μm thickness. Endogenous peroxidase and biotin were blocked. A mouse monoclonal antibody directly against OCT3/4 was used to detect OCT3/4 protein ((1 : 350; SC5279), Santa Cruz, Heidelberg, Germany), which recognises amino acids 1–134 of the protein and therefore recognises OCT4A more specifically than the polyclonal antibody. Expression of OCT3/4 protein was double checked for most samples using a polyclonal antibody ((1 : 350; SC8629), Santa Cruz). Previously (de Jong *et al*, 2005), a similar specificity and sensitivity of these antibodies in GCT tumour diagnostics has been shown, but did not yet differentiate between the different OCT3/4 isoforms. The proteins of the different isoforms only differ at their N terminus. Therefore, the monoclonal antibody is specific for OCT4A. However, strong similarities still exist in these regions between OCT4A and OCT3/4 pseudogenes (Atlasi *et al*, 2008; Wang and Dai, 2010). Slides were incubated as described earlier (Looijenga *et al*, 2003). For different tissues and cell lines known positive controls were used to verify tissue integrity. The following antibodies were used: E-cadherin (1 : 200; clone nch-38, DAKO, Glostrup, Denmark), Ki-67 (1 : 50; clone BIB-1, code M7240, DAKO), AFP (1 : 100; code A008, DAKO), Pankeratin (1 : 400; Cat #MS-743-P, Neomarkers, Fremont, CA, USA), ERG (1 : 100; clone EPR3864, Epitomics, Burlingame, CA, USA), TTF1 (1:200; Cat #MS-699-P, Neomarkers), SOX2 (1 : 250; AF2018, R&D systems, Oxon, UK), NANOG (1 : 400; AF1979, R&D systems), ER (1 : 50); clone 1D5, Neomarkers).

### Statistics

Differences in gene expression between the groups were evaluated using the Mann–Whitney *U*-test, using VassarStats (faculty.vassar.edu). A *P*-value <0.05 was considered as statistically significant. Correlation analysis was performed by calculating the Pearson correlation coefficient using SPSS 15.0.1 (IBM Corp., Armonk, NY, USA). SPSS was also used to design the logistic regression model predicting the presence of a malignant GCT stem cell component based on mRNA expression of the three *OCT3/4* variants.

## Results

### General

Isoform-specific expression of the various isoforms *OCT4A*, *OCT4B* and *OCT4B1* was analysed in a series of UNDIF-GCTs and DIF-GCTs as well as N-GCTs. Moreover, expression of these isoforms was also investigated in a panel of cell lines (both GCT-CL (de Jong *et al*, 2007, 2008a) and N-GCT-CL, the latter including the prostate xenografts). For this purpose, a highly specific set of verified primer pairs was used (see Materials and Methods and Figure 1 for details). The primer pair used to identify *OCT4A* was specifically designed to avoid false-positive results caused by sequence-based similarities between the *OCT4A* transcripts and *OCT3/4* pseudogenes (Atlasi *et al*, 2008; de Jong *et al*, 2008a). The obtained results will be discussed in the following paragraphs for each *OCT4A*, *OCT4B* and *OCT4B1* separately. Subsequently, correlations between the different variants and association with the presence of a malignant GCT stem cell component were investigated. Finally protein expression will be discussed and correlated to mRNA expression.

### OCT4A mRNA expression

**Figure 2:**
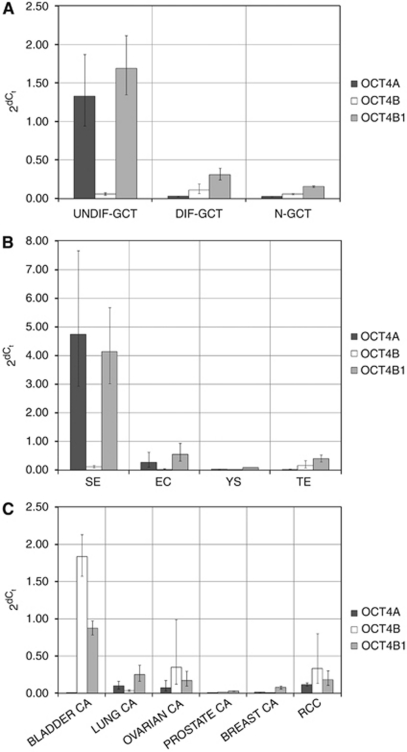
Expression patterns of OCT4A, OCT4B and OCT4B1 in UNDIF-GCT, DIF-GCT and N-GCT. *x* Axis: tumour samples/groups (see Materials and Methods). *y* Axis: 2^d*C*_t_ (normalised to *HPRT*). Error bars depict s.e.m.: variation within the groups (**A**–**C**). (**A**) Average expression for UNDIF-GCT, DIF-GCT and N-GCT groups. (**B**) Average expression for the UNDIF-GCT and DIF-GCT samples grouped per tumour type (SE=seminoma; EC=embryonal carcinoma; YS=yolk sac tumour; TE=teratoma). (**C**) Average expression for the N-GCT samples grouped per tumour type. CA=carcinoma.

*OCT4A* was significantly higher expressed in UNDIF-GCTs than in DIF-GCTs and N-GCTs (Figure 2A, Table 2). There was no significant difference between DIF-GCTs and N-GCTs. An overall higher level of expression was observed in the seminomas when compared with embryonal carcinomas (Figure 2B, Table 2). The DIF-GCTs, that is, yolk sac tumours and teratomas, consistently showed virtually no expression of *OCT4A*. In the N-GCT group, tumours showed no or very low OCT4A expression (bladder-, prostate-, breast-, lung-, ovarian- and renal carcinomas, respectively) (Figure 2C, Table 2).

**Figure 3:**
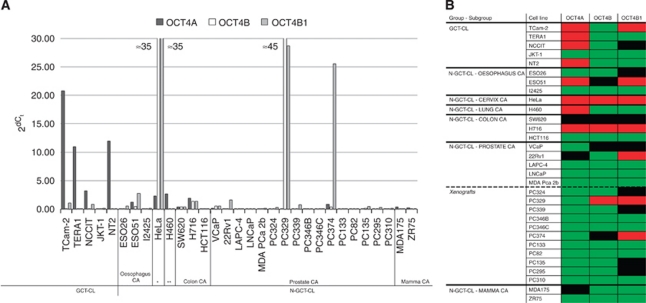
Expression patterns of *OCT4A, OCT4B* and *OCT4B1* in cell lines. (**A**) mRNA levels in all investigated cell lines, both of GCT origin (GCT-CL) and non-GCT origin (N-GCT-CL). ^*^=cervix carcinoma; ^**^=lung carcinoma. *x* Axis: cell lines (see Materials and Methods) and corresponding tumour class. *y* Axis: 2^d*C*_t_ (normalised to *HPRT*). (**B**) Interpretation of the expression of *OCT3/4* isoforms relative to *HPRT*. mRNA expression is scored as high (red, 2^-d*C*_t_ >1), intermediate (black, 2^-d*C*_t_ 0.25-1) or low (green, 2^-d*C*_t_ <0.25). CA=carcinoma. The colour reproduction of this figure is available at the *British Journal of Cancer* online.

OCT4A was significantly higher expressed in GCT-CLs than in N-GCT-CLs (*P*=0.02) (Figure 3). All proven GCT-CLs consistently showed high-expression levels of OCT4A. Expression of OCT4A is known to be absent in JKT-1, a not yet fully classified cell line, suspected to be germ cell-like, although not related to a seminoma (de Jong *et al*, 2007). Of note is that a few N-GCT-CLs showed a relatively high *OCT4A* expression level, defined as at least a ratio of 1.0 when compared with *HPRT.* However, most of the GCT-CLs showed expression levels of >10. Cell lines with ratios of at least 1.0 included ESO51, HeLa, H460 and H716B. Ratios between 0.25 and 1.0 were found in one prostate and one mamma carcinoma cell line (22Rv1 and MDA175). All other N-GCT-CLs showed a low level or absence of OCT4A expression (Figure 3).

### OCT4B mRNA expression

OCT4B was expressed at equally low levels in UNDIF-GCTs and DIF-GCTs (Figure 2A, Table 2). No significant difference was detected between UNDIF-GCTs or DIF-GCTs and N-GCTs (Figure 2A, Table 2). Among the UNDIF-GCTs, seminomas expressed a low level of *OCT4B*, whereas *OCT4B* was virtually undetectable in embryonal carcinoma. The DIF-GCTs showed low expression in teratomas and practically absence of OCT4B in yolk sac tumours (Figure 2B, Table 2). Expression levels of *OCT4B* were highly variable within the N-GCT group. The bladder carcinoma samples showed the highest level of expression, which was rather similar between different samples. The ovarian and renal carcinomas showed an intermediate level of expression, because of a number of high outliers within these groups. Almost no expression was found in lung, prostate and breast cancer samples (Figure 2C, Table 2).

The cell lines showed a highly variable expression of *OCT4B* (Figure 3). All UNDIF-GCT-CLs showed no or a very low level of *OCT4B* mRNA. No significant difference between N-GCT-CLs and UNDIF-GCT-CLs was detected (*P*=0.76). Most of the cell lines showed very low levels or absence of OCT4B expression. Relatively high levels of *OCT4B* were detected in HeLa, as well as in H716 and PC329. Moderate levels were detected in ESO51, VCaP and PC374 (Figure 3).

### OCT4B1 mRNA expression

UNDIF-GCTs showed a significantly higher level of expression of *OCT4B1* than N-GCTs and DIF-GCTs (Figure 2A, Table 2). DIF-GCTs and N-GCTs showed no significant difference (Figure 2A, Table 2). Expression in the UNDIF-GCT group was high in seminoma and intermediate in embryonal carcinoma (Figure 2B, Table 2). Regarding DIF-GCTs, teratomas expressed intermediate levels of *OCT4B1*, whereas expression in yolk sac tumours was low (Figure 2B). Overall, expression of *OCT4B1* was low in N-GCTs. Bladder carcinomas showed, just as in the case of *OCT4B*, a relatively high expression level of *OCT4B1* when compared with other types of N-GCTs (Figure 2C).

Levels of *OCT4B1* expression varied between the cell lines. No significant difference between UNDIF-GCT-CLs and N-GCT-CLs was detected (*P*=0.92). High expression was observed in TCam-2, ESO51, HeLa, H716, 22Rv1, PC329 and PC374. Moreover, intermediate expression levels were present in NCCIT, ESO26, SW620, VCaP, PC324, PC339, PC135 and PC295. In many cases (ESO51, SW620, H716, VCaP, PC329, PC374), intermediate or high levels of OCT4B1 were combined with comparable levels of OCT4B (Figure 3).

During the sequencing process to confirm the PCR products for the different splice variants, a consistent TC insertion was found in exon 2B of *OCT4B1,* being a single nucleotide polymorphism (SNP) (rs34631505). This SNP is located behind the stop codon and therefore has no consequence at the protein level.

### Correlation between mRNA expression of different OCT3/4 isoforms and association of specific isoform expression and presence of a malignant germ cell component

When all samples were combined, OCT4A showed a strong positive correlation with OCT4B1 and a less strong correlation with OCT4B. OCT4B and OCT4B1 did not correlate significantly (Table 3). However, when the samples were split into undifferentiated (UNDIF-GCT) and differentiated tumours (DIF-GCT+N-GCT), strong, positive and highly significant correlations were found between all OCT3/4 variants. Overall, the strength of the correlation approached perfect positive correlation in the UNDIF-GCT. In general, the correlations were less strong in the differentiated tumours, but still highly significant and positive (Table 3). In a binary logistic regression model, OCT4A and OCT4B proved to be significant in predicting the presence of a malignant GCT stem cell component. OCT4A was strongly predictive for the presence a malignant GCT stem cell component (*β*=−4.92, *P*=0.045), whereas OCT4B proved to be suggestive for the absence of such a component, but this association was less strong (*β*=−1.28, *P*=0.048).

### Protein detection

**Figure 4:**
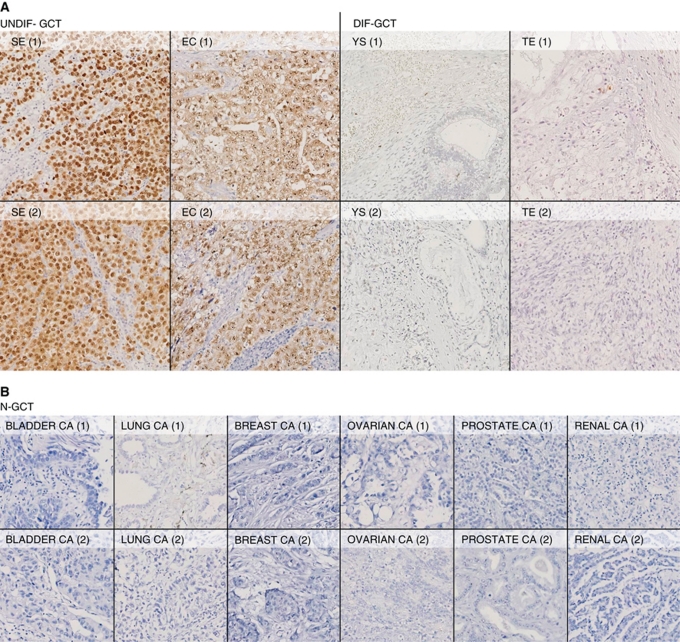
Immunohistochemical detection of OCT3/4 expression in tumour samples. For each tumour type, two different samples are shown. Magnification × 100. (**A**) Protein expression of OCT3/4 in UNDIF-GCTs and DIF-GCTs. Shown are two seminomas (SEs), embryonal carcinomas (ECs), yolk sac tumours (YSs) and teratomas (TEs), of which only the first two types are positive. (**B**) Protein expression of OCT3/4 in N-GCT tumour samples, including two carcinomas of the bladder, lung, breast, ovary, prostate and kidney, respectively, all are negative. All samples were stained using an antibody that is most specific for OCT4A (see Materials and Methods). The colour reproduction of this figure is available at the *British Journal of Cancer* online.

**Figure 5:**
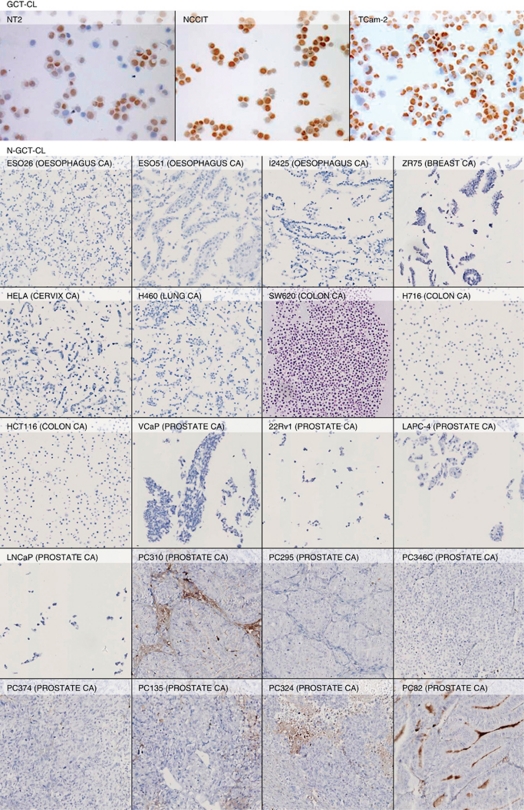
Immunohistochemical detection of OCT3/4 expression in cell lines and xenografts. Stained are the undifferentiated GCT-CLs NT2, NCCIT and TCam-2, which are all positive. All somatic cancer cell lines (ESO26, ESO51, I2425, ZR75, HELA, H460, SW620, H716, HCT116, VCaP, 22Rv1, LAPC-4, LNCaP) and xenografts (PC310, PC295, PC346C, PC374, PC135, PC324, PC82) are negative (some nonspecific staining of necrosis). All samples were stained using an antibody that is most specific for OCT4A (see Materials and Methods). Magnification × 200 GCT-CLs, 100 × N-GCT-CLs. CA=carcinoma. The colour reproduction of this figure is available at the *British Journal of Cancer* online.

Immunohistochemical staining of the various GCT samples and cell lines was performed to assess OCT3/4 protein expression. Clear nuclear staining of tumour cells was shown in UNDIF-GCTs. No expression was detected in the DIF-GCT components or in the N-GCT samples (Figure 4). In accordance with the findings in the tumour samples, nuclear staining of tumour cells was shown in GCT-CLs, while being absent in all N-GCT-CLs and xenografts. Both the EC cell lines (NCCIT and NT2) and the SE cell line TCam-2 were OCT3/4 positive. The nonspecific staining in the xenografts was based on necrosis, and again no cytoplasmic or nuclear staining was detected in these samples (Figure 5). Positive controls for all samples prove that all samples were suitable for immunohistochemistry (Supplementary Figures S1A, B and S4A) and HE staining was used to assess tumour morphology (Supplementary Figures S2A and B). Finally, a double check for OCT3/4 expression was performed by staining the same tumour samples (Supplementary Figures S3A and B) and cell lines/xenografts (Supplementary Figure S4B) with a second (polyclonal) antibody directed against OCT3/4. This confirmed our findings that OCT3/4 protein expression is specific to UNDIF-GCTs and the related cell line models (GCT-CLs). These data are completely in accordance with previously published findings (Looijenga *et al*, 2003; de Jong *et al*, 2005).

## Discussion

### Specificity: the pitfalls of pseudogenes and isoforms

Detection of OCT3/4 pseudogenes can and should be avoided by sufficient DNAse pretreatment of the sample, because the respective sequences might be amplified based on their high level of similarity with the protein-encoding variant (OCT4A) and the absence of introns. Therefore, their amplification in PCR might be falsely interpreted as actual OCT4A expression, suggesting possible translation into OCT4A protein. Moreover, specific PCR primer pairs followed by antibody-based analysis should be used to detect the different isoforms of OCT4 at the mRNA level and the presence of protein (Liedtke *et al*, 2007; Atlasi *et al*, 2008; de Jong *et al*, 2008a; Sotomayor *et al*, 2009).

By using such a validated, isoform-specific primer pair setup, this study shows that OCT4A is highly expressed in UNDIF-GCTs, which are known to have a pluripotent stem cell component, originating from PGCs/gonocytes (Oosterhuis and Looijenga, 2005; Looijenga, 2009). DIF-GCTs and N-GCT show virtually no OCT4A expression, which is in line with the notion that OCT4A is responsible for formation of the protein involved in regulation of pluripotency. In contrast, OCT4B is not differentially expressed between the three groups, whereas OCT4B1 is expressed significantly higher in the UNDIF-GCTs when compared with DIF-GCTs and N-GCTs, as found for OCT4A. These results indeed support the general consensus that OCT4A is the marker for stem cell populations in GCTs, and a similar specificity could be suggested for the OCT4B1 variant. However, OCT3/4 protein is only detected in GCTs and representative cell lines, also in this study. No specific signal could be detected in any of somatic cancers or cell lines investigated, irrespective of mRNA expression pattern. Because OCT4A protein is the only one of the three isoforms that directly regulates pluripotency, expression of this protein is a prerequisite for any cell that uses OCT3/4 as a regulator of pluripotency. Therefore, even specific mRNA detection of OCT3/4 isoforms does not yet conclusively prove the applicability of OCT4A, OCT4B or OCT4B1 in the detection of pluripotent cancer stem cells (see below) or somatic stem cells without protein confirmation.

### Functions of OCT3/4 isoforms

OCT3/4 is a known marker for pluripotency and has been shown to have a role in regulation of pluripotency (Pesce and Scholer, 2000, 2001; Wang and Dai, 2010). Moreover, it is an important diagnostic marker for specific types of GCTs (Looijenga *et al*, 2003; Oosterhuis and Looijenga, 2005; de Jong *et al*, 2005; de Jong and Looijenga, 2006; Looijenga, 2009). Recently, investigations into the broader applicability of OCT4 as a marker in (cancer) stem cell biology showed that only OCT4A (and not OCT4B and OCT4B1) is specific to stem cell (like) populations (Tai *et al*, 2005; Lengner *et al*, 2007; Atlasi *et al*, 2008; Cantz *et al*, 2008; Sotomayor *et al*, 2009; Wang and Dai, 2010). It has been shown that basic levels of *OCT3/4* mRNA (even OCT4A) and expression its pseudogenes, are detectable in somatic (tumour) cells (Wang and Dai, 2010; Zhao *et al*, 2011). However, OCT4A protein expression has so far never been conclusively shown in non-pluripotent cells (Wang and Dai, 2010). In addition, it has been described that OCT4A is primarily localised in the nucleus, whereas OCT4B1 primarily resides in the cytoplasm.

OCT4B might have a role in stress response (Wang and Dai, 2010). The role of OCT4B1 is more elusive. It has been associated with both pluripotency and tumourigenesis (via inhibition of apoptosis and cell cycle deregulation) (Asadi *et al*, 2011). Also, a recent report suggested OCT4B1 to be superior to OCT4A in the detection of stemness, at least in human embryonic stem cells (Papamichos *et al*, 2009). These results are mainly based on statistical correlation and lack a biological explanation as to how OCT4B1 contributes to pluripotency, as this variant cannot be directly translated into a functional transcription factor (Atlasi *et al*, 2008). Moreover, the presence of OCT4B1 expression in cancer tissues, which consists of mainly differentiated tissue, is not satisfactory linked to a specific hypothetical *in situ* population of cancer stem cells. In contrast, Gao *et al*, 2010 conclude that OCT4B1 can be alternatively spliced and subsequently be translated into all OCT4B protein forms , linking OCT4B1 to OCT4B-mediated functions like stress response.

The various OCT3/4 isoforms might also have an integrated function as interchangeable decoys in microRNA (miR)-regulated OCT3/4 protein expression. miRs, specifically miR-145 which targets OCT3/4, have been suggested to have a role in the regulation of pluripotency in general and OCT3/4 translation specifically (Xu *et al*, 2009). Competitive miR binding has been suggested as a biological function of pseudogenes (Poliseno *et al*, 2010). This function might also apply to alternative splice variants that, such as the OCT3/4 variants, share their 3′UTR. OCT4B and/or OCT4B1 might prevent translation inhibition of OCT4A mRNA in stem cell components of GCTs or the other way around in differentiated GCTs or somatic cancer cells. The latter could explain OCT4A mRNA expression without translation into detectable protein in somatic cancer cells. This hypothesis is supported by a correlation analysis on the OCT4A/B/B1 mRNA expression data (Table 3). We showed strong correlations between OCT4B and B1 (Table 3) (expected based on similar function (Gao *et al*, 2010)) and OCT4A and B/B1 (strongest in UNDIF-GCT in which OCT4A is active at the protein level). However, functional studies are required to support this hypothesis.

Finally, individuals homozygous for a polymorphism at the initiating codon of *OCT3/4* (rs3130932) are not able to transcribe OCT4B1, and are therefore lacking the putative encoding proteins (Takeda *et al*, 1992; Hussain *et al*, 2008). Depending on race, minor allele frequencies of 23–33% are reported (Hussain *et al*, 2008). So far, no abnormalities have been found related to the absence of this protein in these individuals, but it would be interesting to investigate the relative frequency of this SNP in GCT patients, specifically with respect to OCT3/4 (protein) expression, tumour characteristics and clinical course.

### OCT3/4 isoforms in (cancer) stem cells

Our results disprove the applicability of OCT3/4 mRNA (Tai *et al*, 2005) for the detection of pluripotent cells (possibly cancer stem cells (Collins *et al*, 2005; Ricci-Vitiani *et al*, 2007)) in solid cancers. Bladder carcinomas showed high mRNA expression of OCT4B and OCT4B1, but no OCT4A. This explains earlier reports of high nonspecific OCT4 expression in this type of cancer (Atlasi *et al*, 2007), but does not indicate the presence of OCT4A-positive cancer stem cells. Moreover, our analysis identified no OCT4A expression in prostate carcinoma, which has been reported before (Sotomayor *et al*, 2009) using a specific primer set (Liedtke *et al*, 2007). In contrast, low, but detectable, levels of OCT4A mRNA were found in lung-, ovary- and renal carcinoma samples. Also, some of the studied cell lines showed OCT4A mRNA expression, suggesting the presence of pluripotent cells in these cultures. However, no OCT3/4 protein expression could be identified in any of the N-GCT samples or N-GCT-CLs, using both monoclonal and polyclonal antibodies. Also the ‘stem cells’ in cell lines do not necessarily represent *in vivo* cancer stem cells, illustrated by the absence of OCT4A mRNA in five lung carcinoma samples and the presence of OCT4A mRNA in lung carcinoma cell line H460.

OCT4A is therefore no marker of cancer stem cell-ness in N-GCT, despite its undisputed crucial role in physiological (maintenance of) pluripotency (in germ cell precursors and their malignant counterparts) (Pesce and Scholer, 2000, 2001). OCT4B1 does have a significant tendency toward specificity for the pluripotent stem cell component of UNDIF-GCTs (Figures 2A and B, Tables 2 and 3). It is however also (highly) expressed in differentiated tumours and cell lines (Figures 2C and 3, Tables 2 and 3). OCT4B1 has been associated with detection of pluripotency before, but no functional relation has been proven, and recent research has functionally linked OCT4B1 to OCT4B (stress response) rather than pluripotency (Gao *et al*, 2010).

## Conclusion

This research confirms that different OCT4 isoforms (and pseudogenes) contribute to nonspecific findings of OCT3/4 expression in various tissues and cell lines. This observation emphasises the necessity of using highly specific primer sets and antibodies to investigate the presence of expression of functional (nuclear) OCT3/4 (protein). The presented data confirms the specificity of OCT4A as a marker for the seminomatous and the stem cell component of non-seminomatous GCTs and illustrates the varying mRNA expression levels of OCT3/4 isoforms in other types of solid cancer and cell lines. OCT4A and OCT4B1 were both confirmed to have a significantly higher expression in tissues with a known stem cell component, but until now, only OCT4A can be directly functionally linked to pluripotency. Moreover, this study shows that OCT3/4 protein detection is of crucial importance, because of clear discrepancies between even isoform-specific mRNA expression and protein detection, possibly due to post-transcriptonal regulation. A synergistic role for the different OCT4 splice variants, possibly by competitive miR binding, might be an interesting model to investigate.

## Figures and Tables

**Table 1:** Samples and cell lines included in the study

**Group**	**Subgroup**	**Cell lines/No. of tumour samples**
Undifferentiated GCT cell lines (UNDIF-GCT-CL)	Seminoma Embryonal carcinoma Other	TCam-2 TERA1, NCCIT, NT2 JKT-1
Non GCT cell lines (N-GCT-CL)	Oesophaguscarcinoma	ESO26, ESO51, I2425
	Cervixcarcinoma	HeLa
	Lungcarcinoma	H460
	Coloncarcinoma	H716, HCT116, SW620
	Prostatecarcinoma	Cell lines: LNCaP, 22Rv1, VCaP, LAPC-4, MDA PCa 2b Xenografts: PC324, PC329, PC339, PC346B, PC346C, PC374, PC133, PC82, PC135, PC295, PC310
	Breastcarcinoma	MDA175, ZR75
Undifferentiated GCTs (UNDIF-GCT)	Seminoma (SE)	5
	Embryonal Carcinoma (EC)	4
Differentiated GCTs (DIF-GCT)	Teratoma (TE)	5
	Yolk sac tumour	1
Non-GCT (N-GCT)	Bladdercarcinoma	5
	Lungcarcinoma	4
	Ovariancarcinoma	5
	Prostatecarcinoma	5
	Rectalcarcinoma	4

**Table 2:**
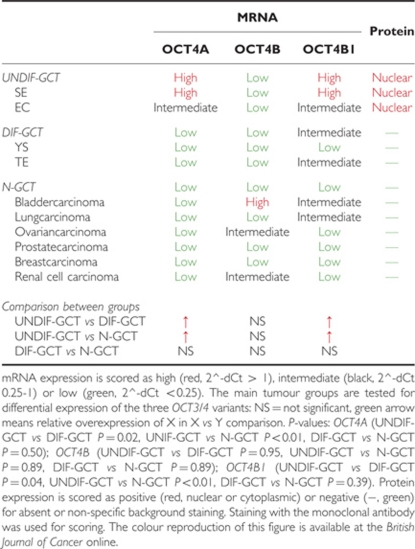
Comparison of OCT4A/B/B1 expression in tumour samples

**Table 3:** Correlation between OCT4A/B/B1 mRNA expression in tumour samples

	**All**		**UNDIF-GCT**		**DIF-GCT+N-GCT**	
	**OCT4B**	**OCT4B1**	**OCT4B**	**OCT4B1**	**OCT4B**	**OCT4B1**
OCT4A	+	+++	+++	+++	++	+++
OCT4B		NS		+++		++

Correlation was assessed using the Pearson correlation coefficient on the mRNA expression data of all tumour samples, the UNDIF-GCT group and the differentiated tumours (DIF-GCT and N-GCT). LEGEND: +++=ρ>0.75; ++=ρ>0.5; +=ρ<0.5; NS=not significant. CORRELATION COEFICIENT/SIGNIFICANCE: All: ρ_*OCT4A,OCT4B*_=0.37 (*P*=0.01), ρ_*OCT4A,OCT4B1*_=0.89 (*P*<0.01), ρ_*OCT4B,OCT4B1*_=0.12 (*P*=0.44). UNDIF-GCT: ρ_*OCT4A,OCT4B*_=0.98 (*P*<0.01), ρ_*OCT4A,OCT4B1*_=1.00 (*P*<0.01), ρ_*OCT4B,OCT4B1*_=0.99 (*P*<0.01). DIF-GCT+N-GCT: ρ_*OCT4A,OCT4B*_=0.63 (*P*<0.01), ρ_*OCT4A,OCT4B1*_=0.97 (*P*<0.01), ρ_*OCT4B,OCT4B1*_=0.68 (*P*<0.01).

## Supplementary Material

Supplementary Figure S1A

Supplementary Figure S1B

Supplementary Figure S2A

Supplementary Figure S2B

Supplementary Figure S3A

Supplementary Figure S3B

Supplementary Figure S4A

Supplementary Figure S4B
