# Specific detection of OCT3/4 isoform A/B/B1 expression in solid (germ cell) tumours and cell lines: confirmation of OCT3/4 specificity for germ cell tumours

**DOI:** 10.1038/bjc.2012.37

**Published:** 2012-02-14

**Authors:** M A Rijlaarsdam, H A D M van Herk, A J M Gillis, H Stoop, G Jenster, J Martens, G J L H van Leenders, W Dinjens, A M Hoogland, M Timmermans, L H J Looijenga

C**orrection to:** *British Journal of Cancer* (2011) **105**, 854–863; doi:10.1038/bjc.2011.270

Upon publication of this paper in Volume 105, the authors noticed an error in the ‘Materials and Methods’ section, under the sub-heading ‘RNA isolation’. An incorrect primer sequence was introduced; we have, therefore, presented the full, corrected paragraph, below.

## RNA isolation

High-quality total RNA was extracted from the abovementioned cell lines and tumour samples using TRIzol Reagent (Invitrogen, Breda, The Netherlands) according to the manufacturer's instructions. Samples were pretreated with DNase I, checked for residual DNA contamination by PCR, after which cDNA synthesis was performed as described before (Looijenga *et al*, 2006; de Jong *et al*, 2008a). For each sample, a no-reverse transcription control was used, and *HPRT* was used as reference level of expression. Quantitative PCR was performed using the Real-Time PCR HT7900 (Applied Biosystems, Foster City, CA, USA). Sequences for the OCT3/4 splice variant specific primers were as described before (Atlasi *et al*, 2008; de Jong *et al*, 2008a). These are highly specific for the different isoforms and even discriminate between OCT4A and its pseudogenes. The following forward (—F) and reverse (—R) primers were used (annotation between brackets=annotation from (Atlasi *et al*, 2008)): HPRT: HPRT244-exon2-F, 5′-AATTATGGACAGGACTGAACGTC-3′ HPRT243-exon3-R, 5′-CGTGGGGTCCTTTTCACCAGCAAG-3′. OCT4A: OCT4A-F (OCT4-AF) 5′-CTTCTCGCCCCCTCCAGGT-3′ OCT4A-R (OCT4-RB1) 5′-AAATAGAACCCCCAGGGTGAGC-3′. OCT4B: OCT4B-F (OCT4-FB) 5′-AGACTATTCCTTGGGGCCACAC-3′ OCT4B-R (OCT4-RB5) 5′-GGCTGAATACCTTCCCAAATAGA-3′. OCT4B1: OCT4B-F (OCT4-FB), 5′-AGACTATTCCTTGGGGCCACAC-3′ OCT4B1-R (OCT-RB4) 5′-CTTAGAGGGGAGATGCGGTCA-3′. The localisation of the different primers is depicted in Figure 1. The efficiency and specificity of these primers was extensively tested before (Atlasi *et al*, 2008). The specificity for human RNA is proven by the absence of any *OCT4*A/B/B1 expression in most of the xenografts, specifically in PC82, which has a large stromal component. Quantitative values were obtained from the Ct. *OCT3/4* mRNAs (A, B and B1) were quantified with relative to *HPRT* (*OCT3/4* mRNA=2^(mean Ct HPRT−mean Ct OCT3/4 (A, B or B1))^) as described before (Livak and Schmittgen, 2001). The *OCT4*B1 PCR products were sequenced using OCT4B1-F and a primer in exon 5 (OCT4B1-R2: (OCT4-RB3) 5′-CCCCCTGTCCCCCATTCCTA-3′) to verify the nature of this splice variant. MicroRNA expression was measured as described previously (Gillis *et al*, 2007).

