# Supplementary figures and images for: Specific detection of OCT3/4 isoform A/B/B1 expression in solid (germ cell) tumours and cell lines: confirmation of OCT3/4 specificity for germ cell tumours
Source: Br J Cancer. 2011 Aug 16;105(6):854–63. doi: 10.1038/bjc.2011.270 (PMC3171004; doi:10.1038/bjc.2011.270)

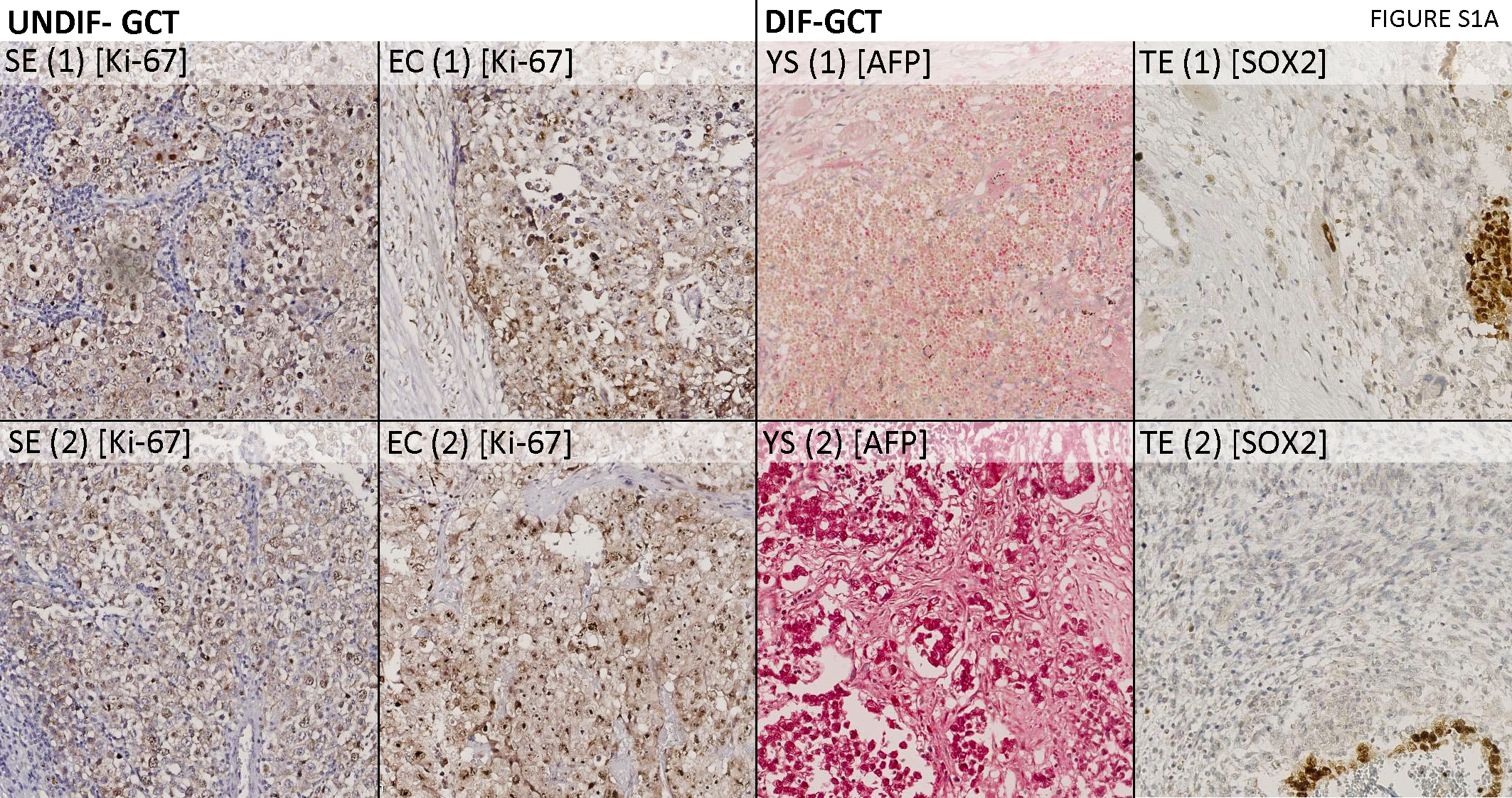

Supplement: Supplementary Figure S1A [file bjc2011270x1.png]

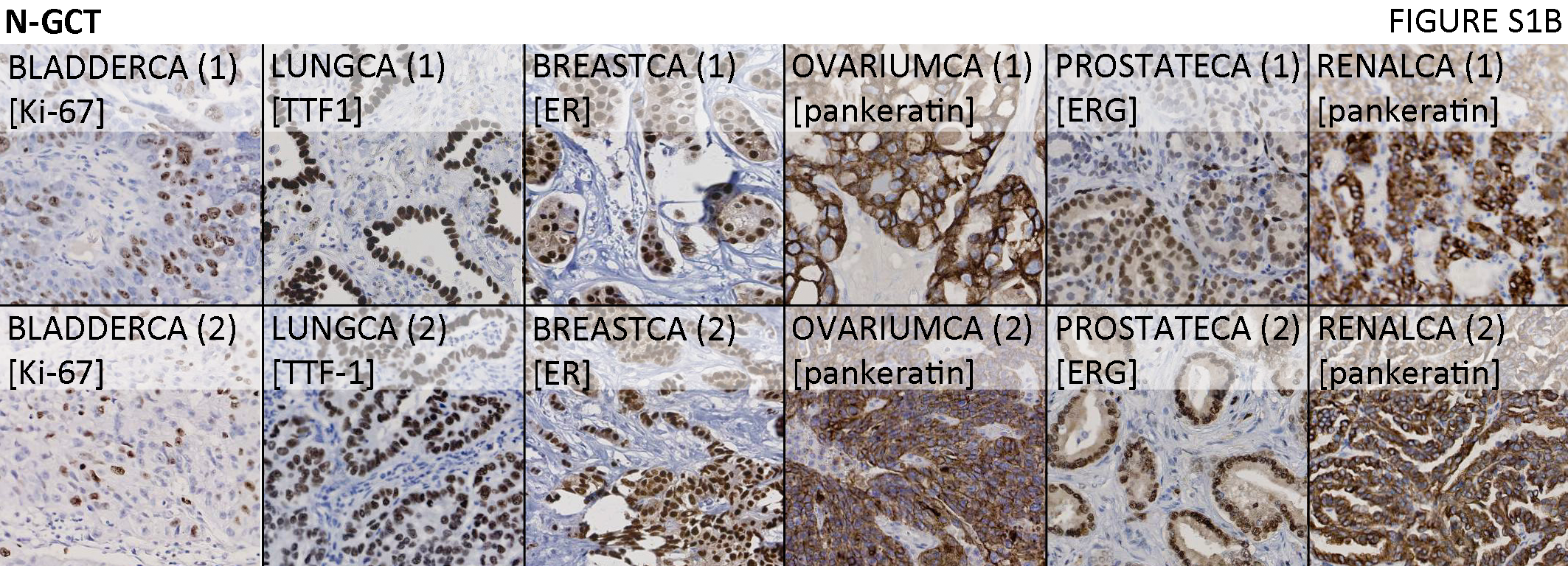

Supplement: Supplementary Figure S1B [file bjc2011270x2.png]

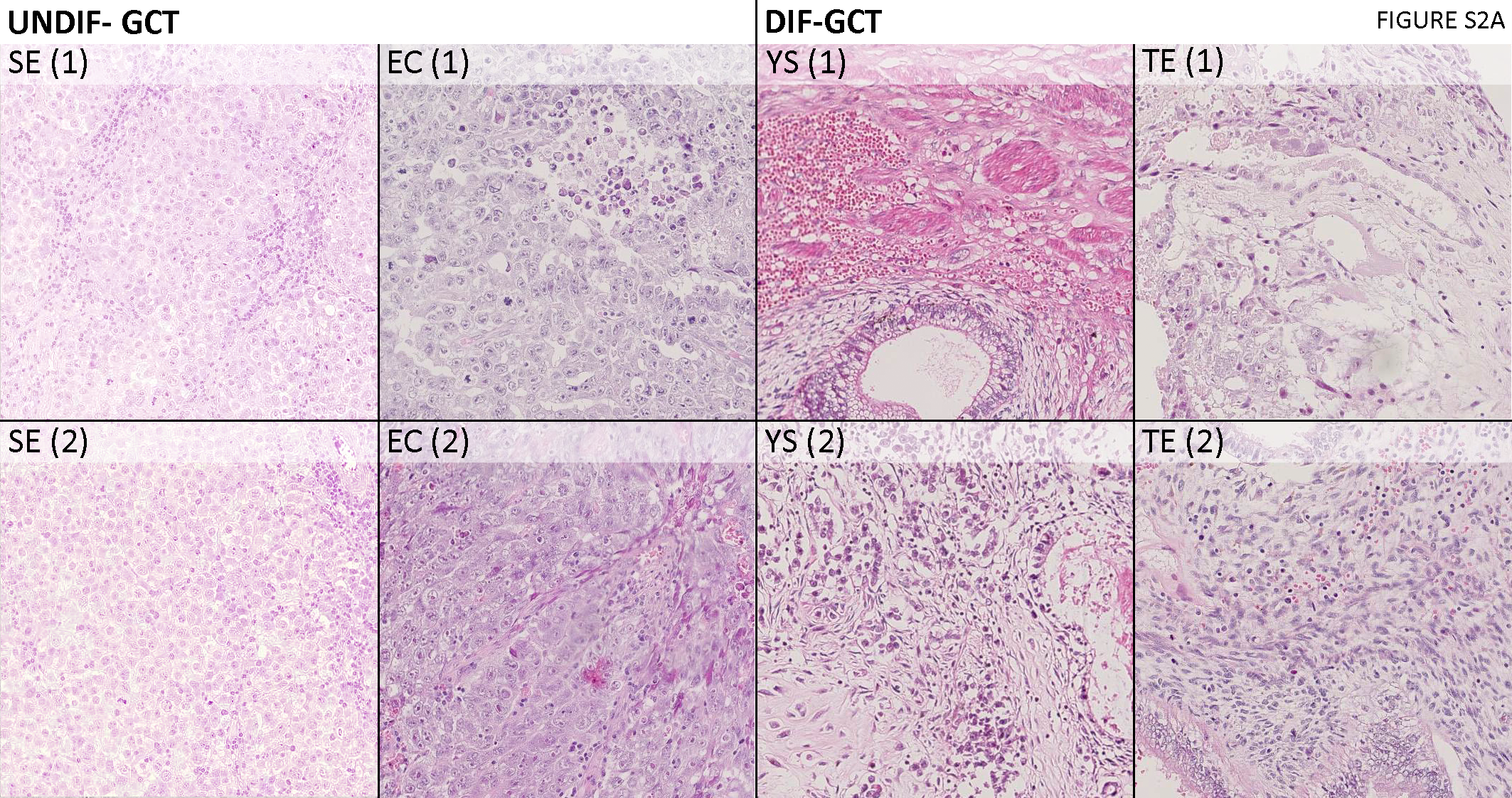

Supplement: Supplementary Figure S2A [file bjc2011270x3.png]

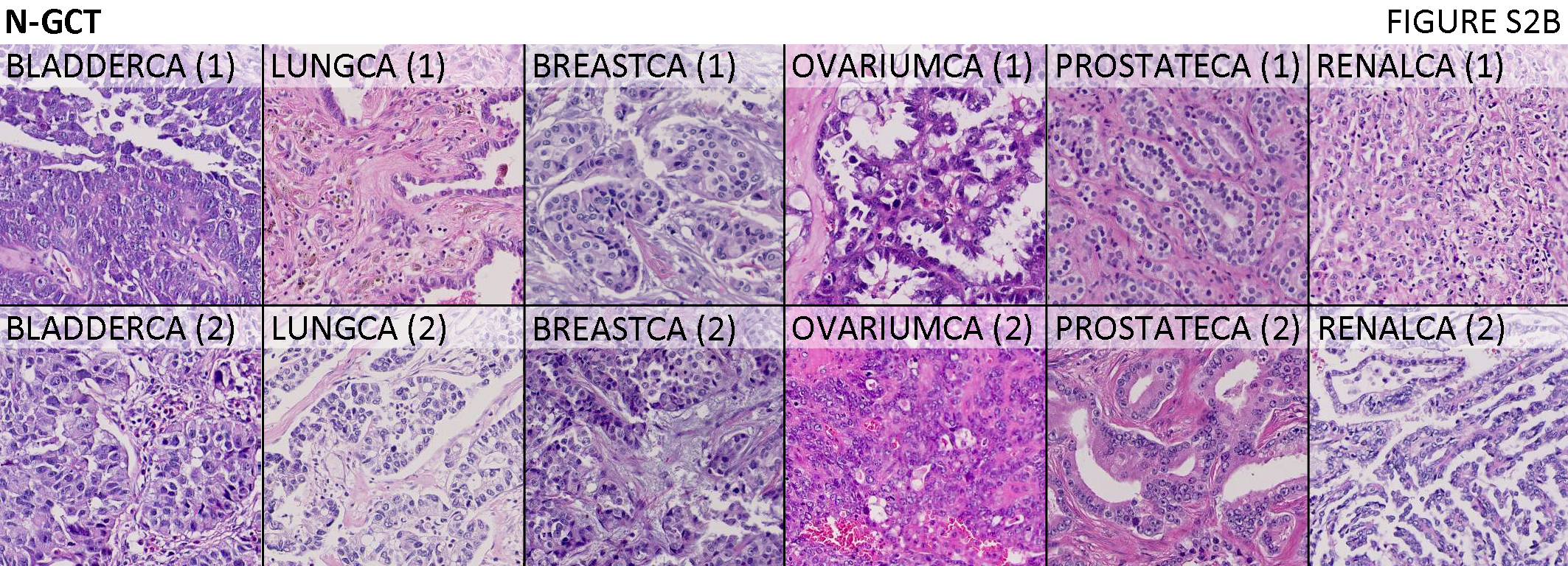

Supplement: Supplementary Figure S2B [file bjc2011270x4.png]

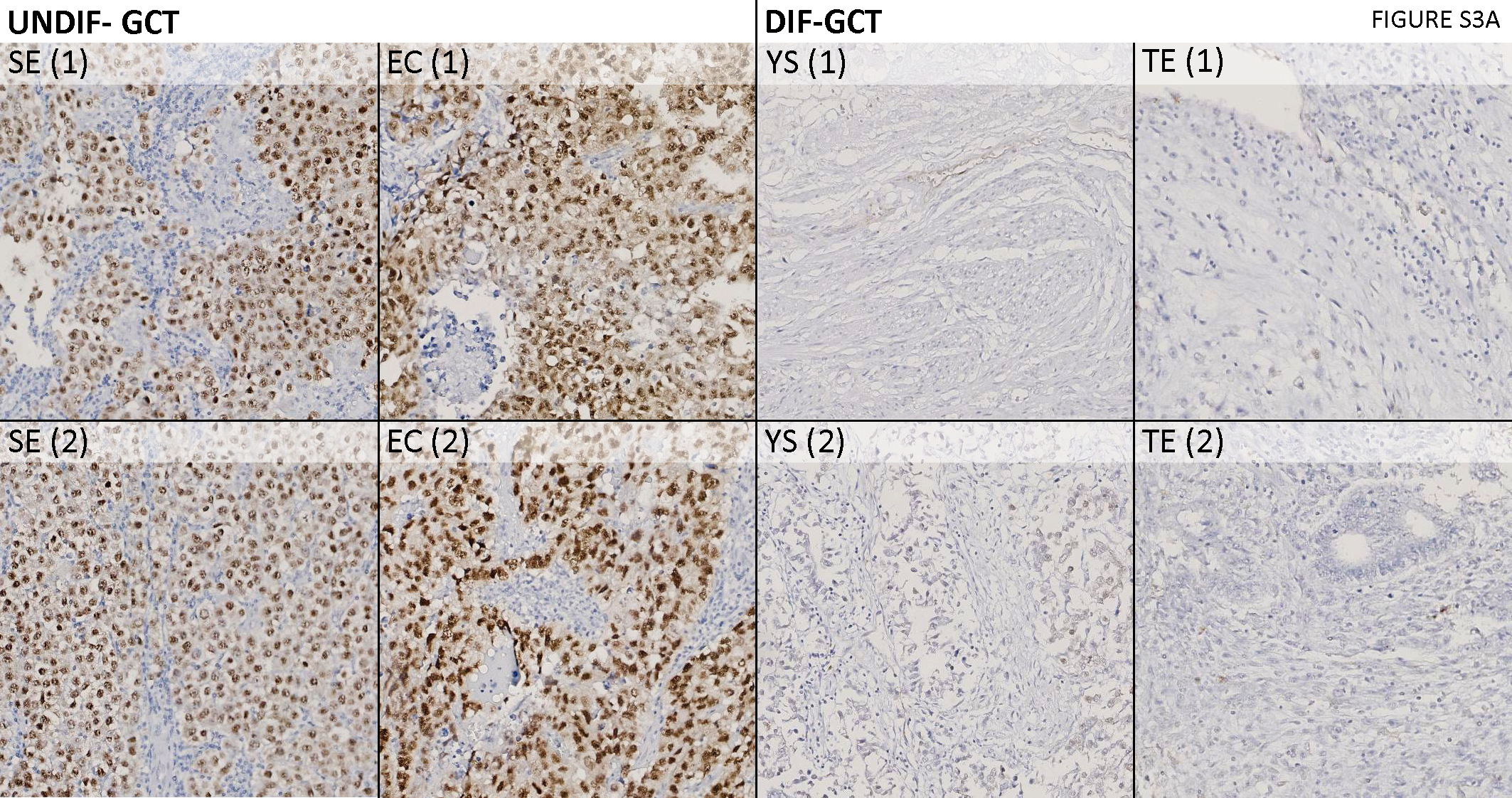

Supplement: Supplementary Figure S3A [file bjc2011270x5.png]

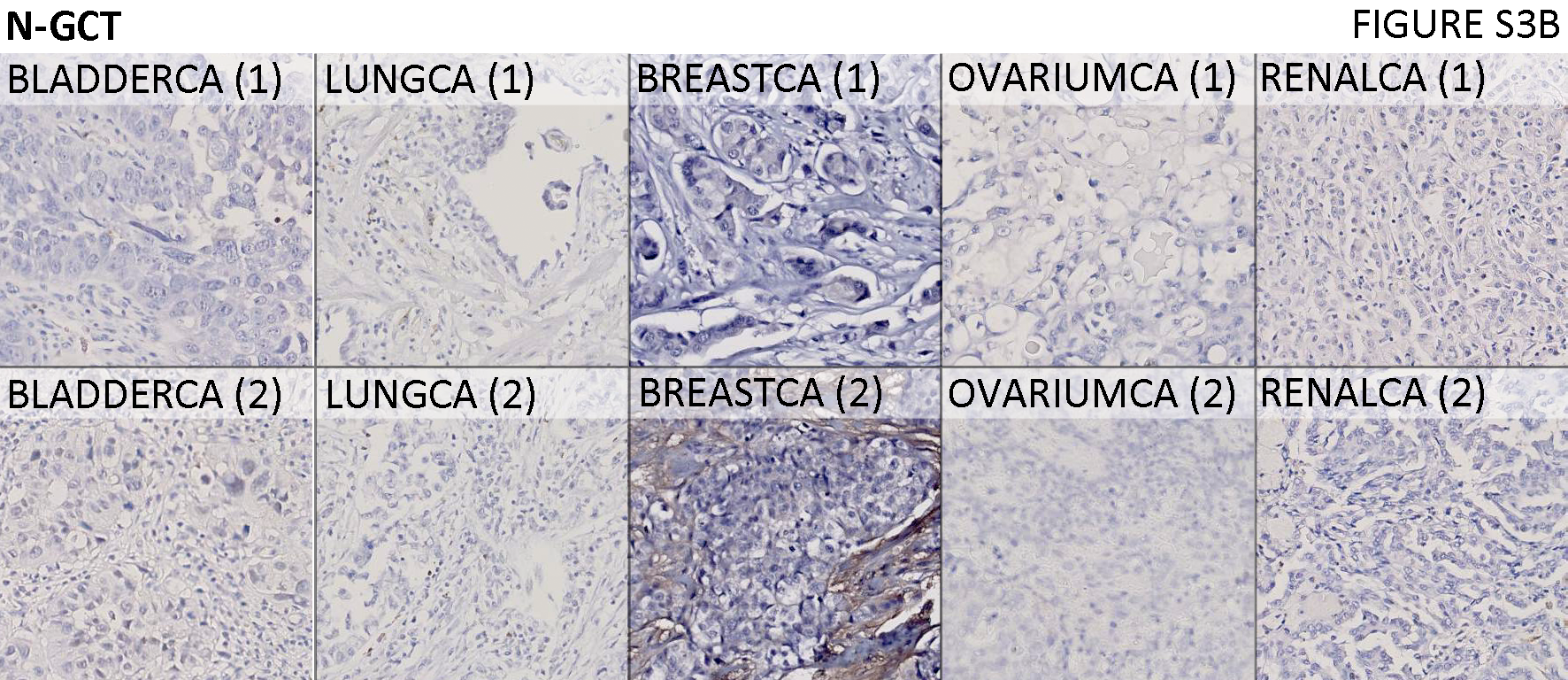

Supplement: Supplementary Figure S3B [file bjc2011270x6.png]

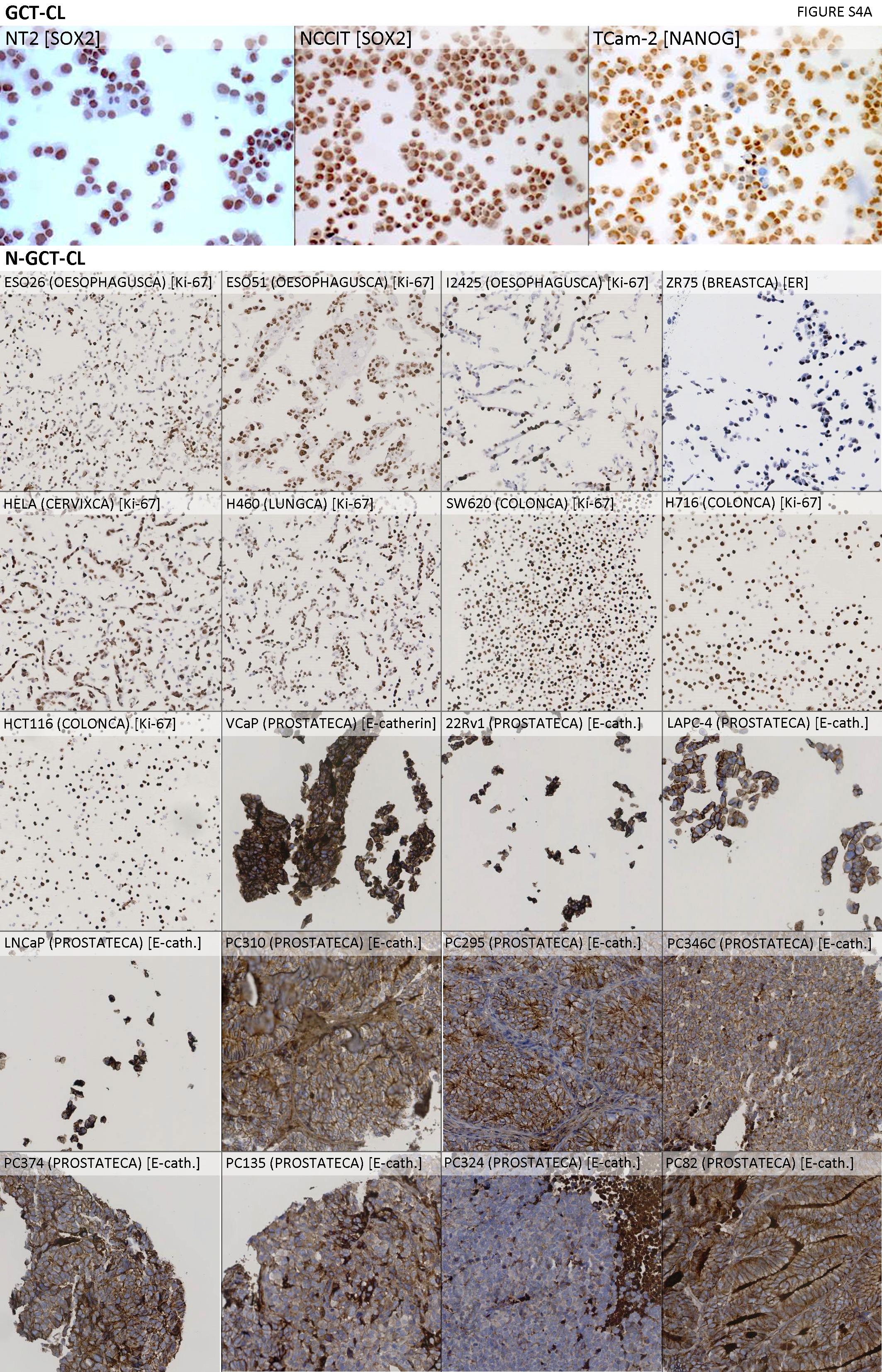

Supplement: Supplementary Figure S4A [file bjc2011270x7.png]

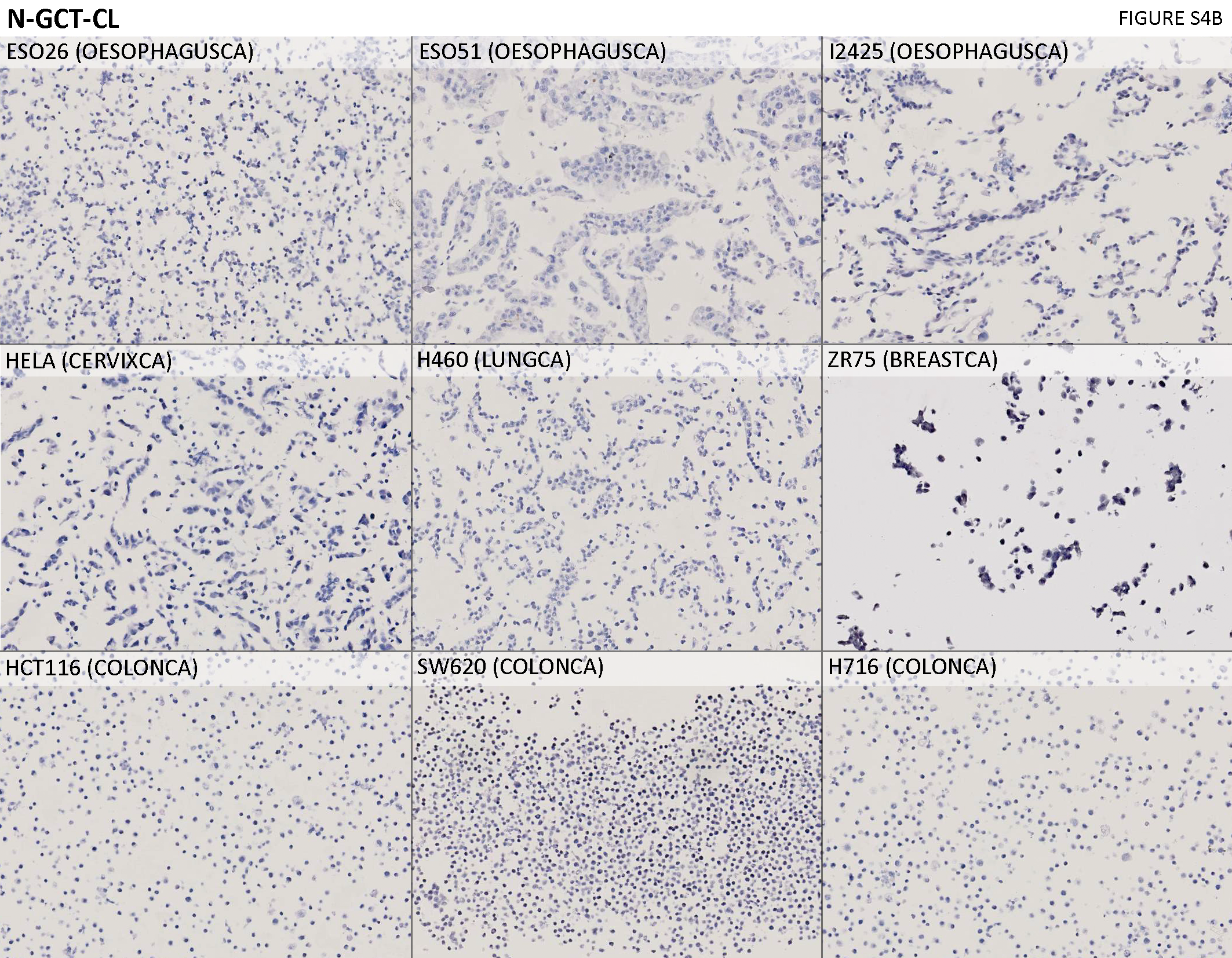

Supplement: Supplementary Figure S4B [file bjc2011270x8.png]
