# Supplementary material for: Specific detection of OCT3/4 isoform A/B/B1 expression in solid (germ cell) tumours and cell lines: confirmation of OCT3/4 specificity for germ cell tumours
Source: Br J Cancer. 2011 Aug 16;105(6):854–63. doi: 10.1038/bjc.2011.270 (PMC3171004; doi:10.1038/bjc.2011.270)
Supplement: Supplementary Figure Legends [file bjc2011270x9.doc]

**Figure S1. Positive controls for immunohistochemistry on GCT and N-GCT samples.** To investigate the tissue integrity for immunohistochemistry, all samples were stained with a known positive control for each tumour type. For each tumour type, two different samples are shown. See legend of Figure 4 for explanation. The protein against which the staining was directed is displayed between brackets. Ki-67: proliferation-related Ki-67 antigen; TTF1: transcription termination factor; ER: estrogen receptor; Pankeratin: staining of fibrous structural proteins (keratin family) present in ovariumcarcinoma; ERG: a transcriptional regulator present in prostate carcinomas. Magnification 100x. (A) GCT samples. The SOX2 positive areas in the TE sections are EC components surrounded by mature teratoma. (B) N-GCT samples. CA = carcinoma.

**Figure S2. Tumor morphology for GCT and N-GCT samples.** Hematoxylin-eaosin staining. Magnification 100x. CA = carcinoma. (A) GCT samples. (B) N-GCT samples. See legend of Figure 4 for explanation.

**Figure S3. Immunohistochemical detection of OCT3/4 expression in tumour samples: polyclonal antibody.** A double check for OCT3/4 expression was performed by staining the same tumour samples with a second polyclonal (less specific) antibody directed against OCT3/4. This antibody is known to display more non-specific background staining. For each tumour type, two different samples are shown. See legend of Figure 4 for explanation. Magnification 100x. CA = carcinoma. (A) Protein expression of OCT3/4 in UNDIF-GCTs and DIF-GCTs. (B) Protein expression of OCT3/4 in N-GCT tumour samples. Prostate carcinoma samples were not included (shortage of material). The staining in breastCA2 is nonspecific, non-nuclear and is absent in the monoclonal staining (Figure 4B).

**Figure S4. Positive controls and confirmation of OCT3/4 expression in cell lines.** (A) Positive controls for immunohistochemistry on GCT-CLs and N-GCT-CLs. To investigate the tissue integrity for immunohistochemistry, all cell lines were stained with a known positive control. See legend of Figure 5 for explanation. The protein against which the staining was directed is displayed between brackets. Magnification 200x GCT-CLs, 100xN-GCT-CLs. (B) Confirmation of OCT3/4 protein expression. A double check for OCT3/4 expression was performed by staining the N-GCT cell lines with a second polyclonal (less specific) antibody directed against OCT3/4. All were confirmed as negative. See legend of Figure 5 for explanation. Prostate carcinoma cell lines/xenografts and GCT-CLs were not included (shortage of material). CA = carcinoma.
